# Mental Health of Canadian Firefighters: The Impact of Sleep

**DOI:** 10.3390/ijerph182413256

**Published:** 2021-12-16

**Authors:** Heidi Cramm, Rachel Richmond, Laleh Jamshidi, Megan Edgelow, Dianne Groll, Rose Ricciardelli, Joy Christine MacDermid, Michael Keiley, R. Nicholas Carleton

**Affiliations:** 1 School of Rehabilitation Therapy, Queen’s University, Kingston, ON K7L 3N6, Canada; rachel.richmond@queensu.ca (R.R.); edgelowm@queensu.ca (M.E.); 2 Department of Psychology, University of Regina, Regina, SK S4S 0A2, Canada; laleh.jamshidi@uregina.ca (L.J.); nick.carleton@uregina.ca (R.N.C.); 3 Department of Psychiatry, Queen’s University, Kingston, ON K7L 3N6, Canada; grolld@queensu.ca; 4 Department of Sociology, Memorial University of Newfoundland, St. John’s, NL A1C 5S7, Canada; rricciardell@mun.ca; 5 School of Physical Therapy and Surgery, Western University, London, ON N6A 3K7, Canada; jmacderm@uwo.ca; 6 Kingston Fire & Rescue, Kingston, ON K7P 1N3, Canada; mkeiley@cityofkingston.ca

**Keywords:** insomnia, shift worker, mental health, public safety personnel

## Abstract

Volunteer and career firefighters are at risk of major depressive disorders, posttraumatic stress disorder (PTSD), alcohol use disorder, and other mental health disorders due to the demanding and unpredictable nature of their employment. The mental health risks are exacerbated by the need to work extended hours, night shifts, and/or rotating schedules, or the competing demands of other employment, especially in volunteer firefighters. The mental health disorders and risk factors interact with altered sleeping patterns. In the current study, we examined volunteer and career firefighters regarding the association between mental health and sleep, drawing from a national Canadian mental health survey of 1217 firefighters. Most (69%) of the firefighters reported less than ideal sleep quality and 21% screened positive for clinical insomnia, with no significant difference between volunteer and career subgroups. Firefighters with insomnia had higher odds ratios (OR) and frequencies for PTSD (OR = 4.98), generalized anxiety disorder (OR = 7.15), panic disorder (OR = 6.88), social phobia (OR = 4.98), and major depressive disorder (OR = 7.91), than firefighters without insomnia. The burden of sleep disorders and their association with mental health disorders suggests that sleep should be considered in health monitoring and self-management, environmental design, fire service work-organization policies, and health programming.

## 1. Introduction

Firefighters are regularly exposed to highly stressful, unpredictable, dangerous, and potentially psychologically traumatic events (PPTE) when responding to calls on duty [1]. Some firefighters classify firefighting as their career, whereas others serve in a volunteer capacity [2]. Most firefighting (83%) in Canada is done by volunteers in rural areas [3] who are on call to provide 24-h emergency services, despite having separate careers [2,4]. Volunteer firefighters respond to overnight calls and are still expected to return to their day jobs, despite these calls impeding their cognitive performance [4]. Although some services maintain 12-h shift schedules, career firefighters typically work on rotating schedules of 24 h on/48 h off, or 48 h on/96 h off [5]; however, some firefighters still adopt a variation of 12, 10, or 8 h shifts [6]. Firefighter shifts, for both career and volunteer workers, can fluctuate frequently between day, night, and 24 h shifts; even a firefighter resting during a 24-h shift with no immediate emergency is still required to be alert, in case of an impending emergency [4,5,7]. Many fire departments allow for career firefighter shifts that last up to 72 consecutive hours [8], increasing firefighters’ susceptibility to shift work health risks. There is an expectation that firefighters sleep during these extended shift call volumes; however, environmental design, staffing levels, seasons and many other factors can interfere with sleep opportunities.

Shift work includes night shifts, rotating schedules, and extended hours [9], and places the worker population at a greater risk of disordered sleep and the subsequent health risks. Surani’s [10] study of individuals from the Detroit general population found evidence that 32.1% of night shift workers and 26.1% of rotating shift workers experienced disordered sleep, compared with only 18% of day workers. Ferri and colleagues [11] found evidence from U.S. nursing data (*n* = 75,000) that death due to cardiovascular incidents was significantly increased for rotating night shift workers. The World Health Organization (WHO) found evidence that circadian rhythm disruption due to shift work is a potential carcinogen, increasing shift workers’ vulnerability to cancer [12]. Additionally, shift workers’ irregular sleeping hours can result in social isolation and generate psychological and psychosocial distress, resulting in an increased risk of the symptoms of mood disorders, anxiety disorders, and alcohol use disorder [13].

Sleep is a vital biological function that directly impacts physical and mental health [14,15]. Most adults (ages 18–60) require a minimum of seven hours of sleep per night that is of good quality, regularity, and appropriate timing to support their physical and mental health [16]. Sleep quality, regularity, and timing help to regulate the circadian rhythm—the body’s biological clock in charge of the sleep wake cycle, autonomic processes, thought processes, emotions and behaviors [9]—and promote sufficient sleep and well-being. Continuous disruption of the circadian rhythm due to irregular sleep stimulates the HPA axis and can lead to an increased stress response and subsequent decline in health [12]. Insufficient sleep (e.g., less than 4–6 h per night) increases the risk of death by suicide [7,17], mortality, cardiovascular disease, depression, diabetes, decreases immune system functioning, and has a negative impact on cognition [14,18]. A large portion of the general U.S. population (35–40%) reports insufficient sleep [14]; similarly, in Europe disordered sleep estimates range from 17% in Italy to 31% in Poland [19].

In addition to dealing with the impact of disordered sleep from shift work, up to 75% of public safety personnel (PSP) are also faced with PPTE on a regular basis [1]. The irregular, lengthy hours of work and increased PPTE exposure increase PSP risk for disordered sleep, and therein their susceptibility to physical and mental health concerns [20]. Firefighters, as a result of their occupations, are also susceptible to musculoskeletal disorders [21], pulmonary conditions [22], cancer [23], and cardiovascular disease [24], all of which reflexively disrupt sleep.

Almost 100% of Canadian firefighters report dozens or hundreds of lifetime exposures to an average of 10.23 different types of PPTE, as a function of their work [1,21,25]. The PPTE firefighters experience have been linked with substantially higher rates of diverse mental disorders in Canada [26] and internationally [27,28], as well as pervasive problems with disordered sleep [29]. When working a 24-h shift, firefighters may be active for the full 24 h, or on alert for emergency calls, neither of which are conducive environments to substantial sleep [7]. The two most commonly reported problems among a sample of U.S. firefighters were that of sleep deprivation at 59% and binge drinking at 58% [7]. Firefighters experience long, irregular shifts, and unpredictable emergencies, all of which are associated with lowered mood, fatigue and alertness, and disordered sleep [7,30]. Disordered sleep also contributed to lower back pain among firefighters in a 13-year longitudinal study [31].

There are extreme physical, psychological and structural demands placed on firefighters, both career and volunteer, necessitating further examination of firefighter job demands, and their impact on sleep and overall health and wellbeing [7]. We have some information connecting firefighters’ sleep to their health, but research is limited, and the connections are not typically specific to firefighters, especially Canadian firefighters. The lack of research is a contributing factor in absent nationwide regulation for the work and rest schedules of Canadian firefighters [8,26]. This study investigated sleep and mental health constructs, such as posttraumatic stress disorder, depression, anxiety, stress, panic, social phobias, and alcohol use.

## 2. Study Aim

The study was designed to examine the association between sleep and mental health among Canadian firefighters.

## 3. Materials and Methods

In the current study, we analyzed data from individuals identifying as firefighters, collected from a larger cross-sectional survey of PSP in Canada [26]. Details of data collection are described elsewhere [26]; in brief, participation was solicited through emails to currently serving PSP employed in communication, correctional, fire, paramedic, or police services. Data in English or French were collected using web-based self-reporting survey methods. The survey included validated screening tools of mental health disorder symptoms, with evidence of diagnostic discriminant validity. Participants were not required to answer any question to proceed through the survey; however, participants were asked to confirm that questions left unanswered were done so intentionally. Participants were asked to provide demographic information including whether they were career or volunteer firefighters, their rank, and their job title. The survey was launched on 1 September 2016, and PSP could participate until 31 January 2017.

### 3.1. Self-Report Symptom Measures

Indications of mental disorder(s) and symptom severity were assessed using well-validated self-report screening tools (described below). These screening tools were not diagnostic; rather, a ‘positive screen’ on any of the tools indicated that an individual reported several symptoms or indicators associated with a disorder.

1. PTSD was assessed using the PTSD Check List 5 (PCL-5) [32]. The PCL-5 is a commonly used 20-item self-report tool that assesses the 20 symptoms of PTSD outlined in the Diagnostic and Statistical Manual of Mental Disorder-fifth edition (DSM-5) (APA, 2013). Individuals were asked to rate how bothersome the 20 items were to them on a scale of 0 (not at all) to 4 (extremely). A total score greater than 32 is considered reasonable for a provisional positive screen for PTSD. Internal consistency of the PTSD check list for the current study was α = 0.95.
2. Major depressive disorder (MDD) symptoms were assessed using the Patient Health Questionnaire 9-item (PHQ-9) [33]. In the PHQ-9 individuals were asked to consider the past two weeks and to rate nine symptoms of depression on a scale of 0 (not at all) to 3 (nearly every day). A provisional screen for MDD was based on 5 of the 9 items being rated at least a 2 or 3, or two questionnaire items; “little interest or pleasure in doing things” and “feeling down, depressed or hopeless” were rated 2 or 3. Internal consistency of the PHQ for the current study was α = 0.90.
3. Panic disorder symptoms were assessed using the Panic Disorder (PD) Symptoms Severity Scale (PDSS) [34]. The PDSS is a 7-item severity scale where items are scored on 5-point scale from 0 to 4. The measure was designed to rate the overall severity of PD symptoms. A total score greater than 9 is considered reasonable for a provisional positive screen for PD. Internal consistency of the PDSS for the current study was α = 0.93.
4. Symptoms of a General Anxiety Disorder (GAD) were assessed using the GAD 7-item Scale (GAD-7) [35]. The GAD-7 is a 7-item questionnaire where individuals are asked to rate how often symptoms of anxiety, such as feeling nervous, anxious, or on edge, have bothered them on a scale of 0 (not at all) to 3 (nearly every day). A total score greater than 9 is considered reasonable for a provisional positive screen for GAD. Internal consistency of the GAD for the current study was α = 0.91.
5. The Depression, Anxiety, and Stress Scale-21 (DASS-21) was also used to measure broad symptoms of depression, anxiety, and stress relative to general population data [36]. The DASS-21 is a 21-item questionnaire divided into three subscales of depression, anxiety, and stress. Items were scored from 0 (does not apply to me at all) to 3 (applies to me very much or most of the time). Internal consistency of depression, anxiety, and stress for the current study was α = 0.86, α = 0.84, and α = 0.83 respectively.
6. Social anxiety disorder (SAD) symptoms were assessed using the Social Interaction Phobia Scale (SIPS) [37]. The SIPS is a 14-item measure of social anxiety symptoms that can be divided into three subscales of Social Interaction Anxiety, Fear of Overt Evaluation, and Fear of Attracting Attention. Subscale scores and an overall score were calculated for the current study. A total score greater than 20 is considered reasonable for a provisional positive screen for SAD. Internal consistency of the SIPS for the current study was α = 0.94.
7. Risky (hazardous) alcohol use with the Alcohol Use Disorders Identification Test (AUDIT) [38]. The AUDIT is consistent with ICD-10 definitions of alcohol use disorder and harmful alcohol use. The AUDIT is a 10-item list of questions relating to an individual’s drinking behavior. Items were scored from 0 (no or never) to 4 (response depends on the question being asked). A total score greater than 15 is considered reasonable for a provisional positive screen for alcohol use disorder. Internal consistency of the AUDIT for the current study was α = 0.82.
8. Sleep disturbance was measured using the Insomnia Severity Index (ISI). The ISI is a self-report questionnaire assessing the nature, severity, and impact of insomnia [39]. Individuals are asked to answer 7 questions rated on a 5-point Likert scale relating to the severity of their sleep difficulties where 0 = no problem; 4 = very severe problem, with total scores ranging from 0 to 28. The total score can then be divided into four categories: absence of insomnia (0–7); sub-threshold insomnia (8–14); moderate insomnia (15–21); and severe insomnia (22–28), or dichotomized into positive screen for clinical insomnia for scores of 15 or greater. Respondents were also asked to rate their sleep quality overall from very poor to very good, and provide an estimate of the number of hours of sleep they get during the workweek (or when they are on shift) and during the weekends (or when they are off shift). Finally, they were asked how many days per week they woke up feeling felt rested. Internal consistency of the ISI for the current study was α = 0.85.

Participants reported their symptoms in the timeframe per the instructions for each scale: PCL-5, past month; MDD, past 14 days; PDSS, past seven days; GAD-7, past 14 days; SIPS, currently, no specific time window; AUDIT, past year; DASS-21, past seven days, and ISI past month.

### 3.2. Statistical Analysis

All data were collected electronically and entered into SPSS v.25 (IBM, New York, NY, USA) for analysis. Missing data were treated as missing, and statistical significance was set as *p* < 0.05. Demographic information such as firefighter current and past employment, age, gender, sleep, and mental health questionnaire scores were described using means, frequencies, percentages, and standard deviations. Overall prevalence estimates for each mental disorder were calculated using (where appropriate) the mean score and the established dichotomous cut-offs.

**Figure 1:**
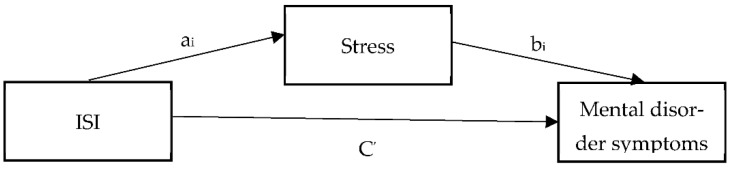
Statistical mediator model sleeping disturbance predicting mental disorder symptoms mediated by stress. Six models were tested with PTSD, PHQ9, GAD7, PDSS, SIPS, or AUDIT as the dependent variable.

The prevalence rates and differences in positive screens for mental disorders between individuals who had screened positive for clinical insomnia and those who had not were calculated using several cross tabulations with χ^2^ tests. For assessing differences between different types of firefighters with respect to the association between sleep disruption and positive screens for mental health disorders, moderator analyses were conducted. A series of logistic regression calculations were then performed to assess the magnitude of differences in mental disorders identified as significantly different between firefighters with and without a positive screen for clinical insomnia, while controlling for age, and gender. The Bonferroni correction was applied to control for multiple comparisons. Pearson correlation analyses were performed between all measures. Finally, six statistical mediation analyses were conducted using PROCESS [40] to assess the mediator effect of stress on the relationship between sleep disturbance and mental disorder symptoms (i.e., PTSD, PHQ9, GAD7, PDSS, SIPS, or AUDIT; Figure 1). Each statistical mediator models included mental disorder symptoms as the dependent variable, stress as the mediator variable, and sleep disturbance as the independent variable.

The study was granted ethical clearance by the University of Regina Institutional Research Ethics Board (File #2016-107). Prior to access to the survey, individuals indicated their willingness to participate by clicking “I agree” at the end of an electronic study information letter.

## 4. Results

A total of 1217 firefighters participated in the survey. Most participants (*n* = 1014; 84%) identified as male, 1086 (89%) were career firefighters, and 141 (11.6%) identified their position as primarily administrative (e.g., Chief, Deputy Chief), 352 (28.9%) as leadership (e.g., chief officers and Captains in emergency response, as well as officers in support divisions such as training, prevention, mechanical, public education, and communications), 310 (25.5%) as a senior firefighter (e.g., First Class), 23 (1.9%) as junior (i.e., 5 to 7 years in career service), and 56 (4.6%) as ‘other’ (e.g., mixed role). A small proportion (*n* = 131, 10.8%) identified themselves as volunteers. See Table 1 for full demographic information. ANOVA produced no statistically significant relationships between positive mental health disorder screenings and the categories of firefighter, or between career and volunteer fighters and positive mental health disorder screenings; as such, the remaining results are for the entire sample of 1217 combined.

Table 2 presents the results of screening for sleep disturbances on the ISI, the perception of overall sleep quality, and the number of hours a night participants sleep when working and when not working. Most respondents (69.2%) reported overall quality of sleep as ‘fair’, ‘poor’, or ‘very poor’, 21.3% screened positive for clinical insomnia, and on average reported waking up feeling rested 2.7 out of 7 days a week.

Table 3 compares the individuals with and without a positive screen for clinical insomnia, on the various screening measures of mental health disorders. The results indicate statistically significant differences between firefighters screening positive for clinical insomnia and those without a positive screen for clinical insomnia, on all mental health disorders except AUDIT.

Table 4 provides odds ratios of screening positive for a mental health disorder for individuals who screened positive for clinical insomnia, controlling for age and gender. Firefighters who screen positive for clinical insomnia have statistically significantly higher odds of screening positive for PTSD, GAD, PD, SAD, and MDD (OR between 4.98 to 8.53; Table 4), a clinically important finding. Moderator analyses produced no statistically significant differences between different types of firefighters on the association between sleep disruption and positive screens for all mental health disorders. The current results suggest different firefighter categories experience similar impacts of positive clinical insomnia on positive screens for mental health disorders.

All correlations between measures were statistically significant, and in the expected direction (Table 5).

Table 6 presents statistical mediation analyses for all models, including total effects, direct effects, and indirect effects of sleep disturbance on mental disorder symptoms with mediation of stress. The results indicated a significant direct effect between sleep disturbance and mental disorder symptoms. Indirect effect of sleep disturbance on PTSD through stress was statistically significant.

## 5. Discussion

The current results suggest a strong association between positive screens for mental health disorders and clinical insomnia. The current results evidenced high rates of sleep disturbance in all firefighter groups, but did not establish differences between different types of firefighters. Firefighters with insomnia also had higher frequencies and odds of screening positive for mental health disorders than those without. There were no statistical differences across firefighter classifications. Most support members (e.g., those involved in training and public education) work regular day hours without shift scheduling; however, to acquire a rank in a support division (e.g., training or fire prevention) requires having been an active emergency response firefighter at some point, either as a volunteer or during career service. Firefighters who respond to emergencies may continue to experience sleep disturbances and concomitant sequelae, well after they change roles.

The current results indicated 69.2% of participants reported having below adequate sleep quality, a finding consistent with that found among Chinese and Iranian (69.9%) [27] and U.S. firefighters (66%) [41], suggesting that this is an ongoing concern for firefighters globally. Disordered sleep increases a person’s risk of suicide, symptoms of major depressive disorder, cardiovascular disease, diabetes, and decreases cognitive functioning [41,42,43]. Shift workers from diverse jobs working nights and rotating shifts have also reported lessened sleep quality and disordered sleep [44]. For shift workers, disordered sleep is explained by the irregularity of work hours and the disruption that these hours have on one’s circadian rhythm [10,44]. Furthermore, similar results have been found among samples of American police officers, both on varied shift work (64.1%) and on day shifts (63.7%) [45], and slightly higher results were found in American paramedics (77%) [46]. The current results evidence poor sleep quality amongst different types of firefighters, regardless of the type of shifts being worked. This is perhaps due to the high-risk behaviors required from firefighters, the unpredictable emergencies they encounter, and their increased exposure to trauma [15]. Different factors may influence sleep in volunteer and career firefighters, but such supposition may require more qualitative investigations.

The current study also evidenced that 21.3% of firefighters had clinical insomnia. An international study on insomnia reported that, relative to health-related quality of life, insomnia is not simply a single-night symptom, but rather a severe condition, of which the impact on one’s health-related quality of life can be compared to diabetes and major depressive disorder [47]. The current study also evidenced that firefighters with a positive screen for clinical insomnia were much more likely to screen positive for mental health disorders than firefighters that had a negative screen for insomnia. The firefighters with insomnia had higher positive screening frequencies for PTSD, MDD, GAD, SAD, PD, and risky alcohol use than firefighters without insomnia. The firefighters also had higher odds of screening positive for PTSD, GAD, PD, SAD, and MDD than their counterparts without insomnia. In addition to the GAD symptoms and lower quality of life that accompanies insomnia, Sing and Wong [48] found that it also has a mediating effect on the optimism associated with MDD. This implies that those with insomnia symptoms have less optimism towards their depression, which furthers their negative health path [48]. Firefighters are already predisposed to challenging mental disorder symptoms, due to the realities of their occupation [1,49], but insomnia serves as an exacerbating factor associated with decreased overall health optimism, which can further elevate risks to their health.

Researchers have documented insomnia as a comorbidity and a precursor to mental health disorders [20,50,51]. These findings in addition to those of the current study make a case for early intervention regarding sleep disturbances such as insomnia. A sleep health program which included three components—mandatory educational sessions, voluntary sleep disorders screening, and sleep disorders diagnosis and treatment for those who screened at risk for a sleep disorder—was implemented for U.S. firefighters [52]. Those that attended the educational sessions took 46% fewer days off due to disability and were 24% less likely to file an official injury report than the controls. An intervention program using behavior and imaging therapy found significant improvements in sleep, as well as symptoms of MDD and PTSD in firefighting participants [53]. Targeting disordered sleep in firefighters may result in fewer comorbidities and the prevention of numerous mental health disorders linked to insomnia (e.g., MDD, GAD, PTSD) [20,52,53,54]. Career, volunteer, and paid on-call firefighters all have different vocational demands that differentially impact their work-life balance [55]; accordingly, each group may need tailored programming to support their health.

There may also be opportunities to develop organization-level modifications for work that lessens the alerting impact of fire calls on firefighters. Given it is adaptive and essential that firefighters learn to rest in a state of readiness, the longer-term impacts on their capacity to enjoy restful and restorative sleep are unclear. The expectation of having sleep interrupted by an alarm, and the need to urgently mobilize results in “light” sleep that is perceived as poor, from which one is easily woken [55]. A recent study compared cortisol levels for day and night alarms among firefighters, reporting that higher cortisol levels during night alarms may be due to a reduced ability for firefighters to predict when a call comes in, and a louder noise relative to the reduced background noise [56]. There is limited but emerging research that suggests adapting the alerting methods to a “ramp-up” tone; limited to station tasks, this response to a given call can reduce the impact on firefighter heart rate throughout their work days [57]. Such interventions may offer some preventive benefit, especially to firefighters with PPTE and whose elevated acoustic startle responses suggest vulnerability for posttraumatic stress [58]. Other studies have demonstrated that the “alarm stimulus signals the potential for exposure to trauma” [55], suggesting researchers should investigate how the physiological and psychological responses to alarms impact firefighters, their sleep, and their mental health.

## 6. Limitations of the Study

The sample size of the volunteer firefighters was relatively small, as was the number of women firefighters, which limited our ability to nuance sex or gender-based differences, limiting our confidence that these results apply to women and/or volunteer firefighters. The data were also derived from self-report surveys, which means personal and societal biases may have influenced the results. Due to these measures, participants were also required to remember and report their sleep and mental health data from their current state to the past year, which may have created recall biases. These limitations were offset by the use of screening tools with evidence of diagnostic discriminant validity. Another limitation is that we did not objectively assess the shift schedule these firefighters were on. Although firefighters generally operate within a shift-based schedule which influences their sleep, we know that these can differ throughout different regions across the country. Future research should investigate the influence of differing shift schedules on sleep and mental health disturbances. Additionally, as this study was cross sectional, the results should be interpreted with caution and no causal inferences should be made. In this study we were not able to assess the direction of the relationship between sleep and mental health disorders. Researchers have explained that the relationship between sleep disturbances and mental health may be bidirectional [59]. Future research should utilize a longitudinal approach in order to understand the directionality of this relationship for Canadian firefighters.

## 7. Conclusions

Many participants (21%) screened positive for clinically significant insomnia. Canadian firefighters report mental health disorders at a prevalence rate of 20.2% [26], which is approximately twice that of the general Canadian population. In the current study, participating firefighters who screened positive for insomnia were statistically significantly more likely to screen positive for PTSD, GAD, PD, SAD, and MDD (OR between 4.98 to 8.53) (Table 4). Future researchers should explore the differences in the sleep and health between volunteer and professional firefighters. Insomnia is associated with several comorbidities and firefighters are already at increased risk, relative to the general population; as such, tailored interventions to improve the sleep of firefighters and other PSP should be developed and assessed by future researchers.

## Figures and Tables

**Table 1:** Demographic Characteristics of the Study Sample.

Sociodemographic Variables	*N*	%
Sex		
Male	1024	84.1
Female	77	6.3
Age		
18–29	48	3.9
30–39	248	20.4
40–49	382	31.4
50–59	361	29.7
60 and older	64	5.3
Marital status		
Married/Common-law	896	73.6
Single	84	6.9
Separated/Divorced/Widowed	79	6.2
Re-married	36	3.0
Province of Residence		
Western Canada (BC, AB, SK, MB)	609	50.0
Eastern Canada (ON, QC)	495	40.7
Atlantic Canada (PEI, NS, NB, NFL)	58	4.8
Northern Territories (YK, NWT, NVT)	2	0.2
Ethnicity		
Caucasian	1027	84.4
Other	72	5.9
Education level		
High school or less	146	12.0
Some post-secondary (less than 4-year college/university program)	648	53.2
University degree/4-year college or higher	281	23.1
First spoken language		
English	959	78.8
French	100	8.2
Other	39	3.2
Years of service		
More than 16 years	839	68.9
10 to 15 years	215	17.7
5 to 9 years	111	9.1
Less than 4 years	52	4.3
Category of Service		
Fire Administration	141	11.6
Fire Leadership	352	28.9
Sr Firefighter	310	25.5
Jr Firefighter	23	1.9
Other	56	4.6
Not provided	335	27.5
Career or Volunteer		
Career	1086	89.2
Volunteer	131	10.8

**Table 2:** Self-reported sleep quality.

	All Firefighters *n* = 1217	
	*N*	%
ISI Score interpretation ^1^		
No clinically significant insomnia	284	37.4
Subthreshold insomnia	314	41.3
Clinical insomnia (moderate)	140	18.4
Clinical insomnia (severe)	22	2.9
Screen positive for clinical insomnia (yes)	162	21.3
How would you rate your quality of sleep overall?		
Very poor	46	6.0
Poor	196	25.7
Fair	287	37.6
Good	186	24.3
Very good	49	6.4
	M Hours	SD
On average, how many hours per night do you sleep on weeknights or when you are working shifts?	6.3	1.3
On average, how many hours per night do you sleep on weekends or when you are not working shifts?	7.0	1.5
On average, how many days per 7-day week do you wake up feeling rested?	2.7	2.1

^1^ ISI = Insomnia severity index. Percentages reflect the percent of those answering the question.

**Table 3:** Frequency and percent of individuals who screen positive for symptoms of the following mental health disorders with and without screening positive for insomnia.

	Negative Screen for Clinical Insomnia *n* = 598		Positive Screen for Clinical Insomnia *n* = 162		Chi-Square
	*N*	%	*n*	%	
PTSD ^1^ (PCL-5) ^2^	43	7.2	46	28.4	55.29 ***
Depression (PHQ-9) ^3^	65	10.9	81	50.0	124.03 ***
Anxiety (GAD-7) ^4^	33	5.5	49	30.2	80.98 ***
Social anxiety disorder (SIPS) ^5^	34	5.7	45	27.8	66.79 ***
Panic disorder (PDSS) ^6^	21	3.5	32	19.8	52.84 ***
Risky Alcohol Use (AUDIT) ^7^	42	7.8	18	12.1	2.63
DASS Depression ^8^	75	12.5	75	46.3	92.46 ***
DASS Anxiety	140	23.4	102	63.0	91.32 ***
DASS Stress	27	4.5	46	28.4	83.36 ***

^1^ PTSD = posttraumatic stress disorder; ^2^ PCL-5 = posttraumatic stress disorder checklist for DSM-5; ^3^ PHQ-9 = patient health questionnaire; ^4^ GAD-7 = generalized anxiety disorder scale; ^5^ SIPS = social interaction phobia scale; ^6^ PDSS = panic disorder symptoms severity scale; ^7^ AUDIT = alcohol use disorders identification test; ^8^ DASS = depression, anxiety, and stress symptoms (DASS-21); *** *p* ≤ 0.001.

**Table 4:** Odds ratios and 95% CI of differences in symptoms of mental disorders in individuals who screened positive for clinical insomnia controlling for age and gender. Reference category is no positive screen (OR = 1.00).

Mental Health Measure	Positive Screen for Clinical Insomnia Odds Ratio (95% CI)	*p*-Value for Moderator Analysis
PTSD ^1^ PCL-5 ^2^	4.98 (3.11, 7.96) ***	>0.05
DASS depression ^3^	6.11 (4.11, 9.10) ***	>0.05
DASS anxiety	5.40 (3.72, 7.85) ***	>0.05
DASS stress	8.53 (5.07, 14.36) ***	>0.05
GAD-7 ^4^	7.15 (4.38, 11.69) ***	>0.05
PHQ-9 ^5^	7.91 (5.27, 11.87) ***	>0.05
PDSS ^6^	6.88 (3.81, 12.42) ***	>0.05
SIPS ^7^	6.32 (3.83, 10.41) ***	>0.05
AUDIT (risky drinking) ^8^	1.57 (0.87, 2.84)	>0.05

^1^ PTSD = posttraumatic stress disorder; ^2^ PCL-5 = posttraumatic stress disorder checklist for DSM-5; ^3^ DASS = depression, anxiety, and stress symptoms (DASS-21); ^4^ GAD-7 = generalized anxiety disorder scale; ^5^ PHQ-9 = patient health questionnaire; ^6^ PDSS = panic disorder symptoms severity scale; ^7^ SIPS = social interaction phobia scale; ^8^ AUDIT = alcohol use disorders identification test; *** *p* ≤ 0.001.

**Table 5:** Correlations between measures.

Measure	Insomnia Severity Index (ISI)	PTSD	PHQ-9	GAD-7	SIPS	PDSS	AUDIT	DASS Depression	DASS Anxiety
PTSD	0.52 **	1							
PHQ-9	0.64 **	0.74 **	1						
GAD-7	0.53 **	0.74 **	0.80 **	1					
SIPS	0.39 **	0.44 **	0.51 **	0.54 **	1				
PDSS	0.35 **	0.62 **	0.59 **	0.66 **	0.47 **	1			
AUDIT	0.22 **	0.20 **	0.29 **	0.21 **	0.11 **	0.18 **	1		
DASS Depression	0.52 **	0.77 **	0.85 **	0.81 **	0.50 **	0.63 **	0.26 **	1	
DASS Anxiety	0.48 **	0.76 **	0.78 **	0.80 **	0.54 **	0.65 **	0.22 **	0.88 **	1
DASS Stress	0.53 **	0.76 **	0.82 **	0.80 **	0.50 **	0.65 **	0.25 **	0.90 **	0.87 **

PTSD = posttraumatic stress disorder; DASS = depression, anxiety, and stress symptoms (DASS-21); GAD-7 = generalized anxiety disorder scale; PHQ-9 = patient health questionnaire; PDSS = panic disorder symptoms severity Scale; SIPS = social interaction phobia scale; AUDIT = alcohol use disorders identification test; ** *p* ≤ 0.01.

**Table 6:** Mediation analyses examining direct and indirect effects of sleep disturbance on PTSD, PHQ9, GAD7, PDSS, SIPS, or AUDIT symptoms through stress.

		Total Effect (*c*)				Direct Effect (*c’*)			
Outcome (Y)		Coefficient		SE		Coefficient		SE	
PTSD		1.43 ***		0.09		0.43 ***		0.08	
PHQ-9		0.61 ***		0.03		0.27 ***		0.02	
GAD-7		0.41 ***		0.02		0.11 ***		0.02	
SIPS		0.63 ***		0.05		0.28 ***		0.06	
PDSS		0.22 ***		0.02		−0.01		0.02	
AUDIT		0.20 ***		0.03		0.10 **		0.04	
						**Indirect Effect (*a_i_b_i_*)**			
	**Direct Effect (*a_i_*)**		**Direct Effect (*b_i_*)**					**95% CI**	
**Mediator**	**Coefficient**	**SE**	**Coefficient**	**SE**	**Outcome (*Y*)**	**Coefficient**	**Boot SE**	**Lower**	**Upper**
Stress	0.72 ***	0.04	1.40 ***	0.06	PTSD	1.00	0.08	0.84	1.16
Stress	0.71 ***	0.04	0.47 ***	0.02	PHQ-9	0.34	0.03	0.28	0.40
Stress	0.71 ***	0.04	0.42 ***	0.01	GAD-7	0.30	0.03	0.25	0.36
Stress	0.71 ***	0.04	0.49 ***	0.04	SIPS	0.35	0.05	0.25	0.46
Stress	0.73 ***	0.04	0.30 ***	0.02	PDSS	0.22	0.03	0.17	0.27
Stress	0.73 ***	0.04	0.13 ***	0.03	AUDIT	0.09	0.02	0.05	0.14

PTSD = posttraumatic stress disorder; DASS = depression, anxiety, and stress symptoms (DASS-21); GAD-7 = generalized anxiety disorder Scale; PHQ-9 = patient health questionnaire; PDSS = panic disorder symptoms severity scale; SIPS = social interaction phobia scale; AUDIT = alcohol use disorders identification test; CI: confidence interval; ** *p* ≤ 0.01, *** *p* ≤ 0.001.

## Data Availability

The analyzed data were derived from a larger Canadian study [26]. No new data were created or analyzed in this study. Data sharing is not applicable to this article.
